# Supplementary material for: Comparison of Pan-Lyssavirus RT-PCRs and Development of an Improved Protocol for Surveillance of Non-RABV Lyssaviruses
Source: Viruses. 2023 Mar 4;15(3):680. doi: 10.3390/v15030680 (PMC10052027; doi:10.3390/v15030680)
Supplement: Supplementary file 1 [file viruses-15-00680-s001.zip › viruses-2175311-Supplementary Material S2; Figure S1; Figure S2.pdf]

Supplementary Material S2

Preparation of in Vitro Transcribed RNA Samples

Plasmids containing lyssavirus genome sequences encompassing the assay target regions were produced either in house using the pCR™II Vector (TA Cloning™ Kit, Dual Promoter, Invitrogen, Massachusetts, United States) as described previously [10,25] or purchased from Vector Builder (Neu-Isenburg, Germany) based on reference sequences available in GenBank. To produce plasmids *de novo*, we used genomic RNAs extracted from lyssavirus isolates using the NucleoSpin™ RNA (Macherey-Nagel™, Germany) or the QIAamp® Viral RNA Mini kit (QIAGEN, Hilden, Germany) according to the manufacturer's instructions. In order to obtain entire 3'UTR and nucleocapside genome, the total RNAs were ligated to DNA oligonucleotide as described elsewhere [44], and amplified employing forward primer (5′-CAGCGAC-CGTTCGATCGC-3′) and strain-specific reverse primer targeting phosphoprotein (sequences available upon request). After gel electrophoresis, bands of the expected size were purified using the NucleoSpin® Gel and PCR Clean-up kit (Macherey-Nagel), and then cloned into the pCR™II Vector (TA Cloning™ Kit, Dual Promoter, Invitrogen, Massachusetts, United States). Ligation reactions were used to transform DH5α competent cells (ThermoFisher Scientific). Ampicillin-resistant colonies were screened with FW/REV M13 primers (5′-GTAAAACGACGGCCAG-3 and 5′-CAGGAAACAGCTATGAC-3′) flanking the multiple cloning site. Candidate positive clones were further propagated for subsequent plasmids purification using the GenElute™ Plasmid Miniprep Kit (Merck) and Sanger sequencing to verify the absence of mutations within the inserts. To produce DNA template for *in vitro* transcription reactions, plasmids containing lyssavirus target sequences were amplified using M13 primers, purified in agarose gel and sequenced to verify sequence identity. The concentration of DNA was measured by spectrophotometry (NanoDrop™ Lite Spectrophotometer). Purified DNA was transcribed to RNA using the MEGAscript™ T7 Transcription Kit (Invitrogen) and subsequently and incubated with TurboDNase for 15 min at 37 °C to remove DNA residuals. RNAs were purified with the MEGAclear™ Transcription Clean-Up Kit (Invitrogen) according to the manufacturer's recommendations. RNA purity was confirmed by both Nanodrop spectrophotometric (NanoDrop™ Lite Spectrophotometer, Thermo Fisher Scientific) reading ratio (260/280) and Agilent analysis (Agilent RNA 6000 Nano kit), yielding a unique and neat peak of the expected size for all the RNAs. RNA concentration was assessed by fluorometry (Qubit™ RNA High Sensitivity kit and Qubit 4 Fluorometer, Invitrogen) applying the following formula:

$$\boldsymbol{copy\ number} = \frac{\boldsymbol{M}\ (\text{concentration of RNA (g/ml)} \times \boldsymbol{N}\ (\text{Avogadro's number} = 6.02 \times 10\text{^}23\ \text{molecole/mol})}{\boldsymbol{L}\ (\text{number of bases which depends on strain; Kb}) \times \boldsymbol{D}\ (\text{factor of dilution} = 3.41\ \text{x}\ 10\text{^}5\ \text{g/mol/Kb})}$$

In vitro transcribed RNA was normalized to 1×10^{10} RNA copies/µl, aliquoted, supplemented with 40 U of RNasin Plus RNase Inhibitor (Promega) and stored at ≤−70°C until use.

Protocol of (n) LN34 rRT-PCR

Amplification reaction was carried out using Ag-Path ID One-Step RT-PCR Kit (Life Technologies) on a CFX 96 Deep well Real-Time PCR System, C1000 Touch (Biorad) in a final volume of 25 µl with 800 nM each primer (FW1-5 and REV 2-3) and 200 nM each probe (LN34 and LN34probe). Thermal profile was as follows: 50°C for 30 min, 95°C for 10 min, followed by 45 cycles at 95°C for 15 sec and 56°C for 30. Data were analyzed using the Bio-Rad CFX Manager software (Version 3.1), with a threshold set manually above the background noise (approximately 100 RFU) while taking in consideration Ct values of artificial positive RNA (ranging from 21-24 Ct) published in original protocol [26].

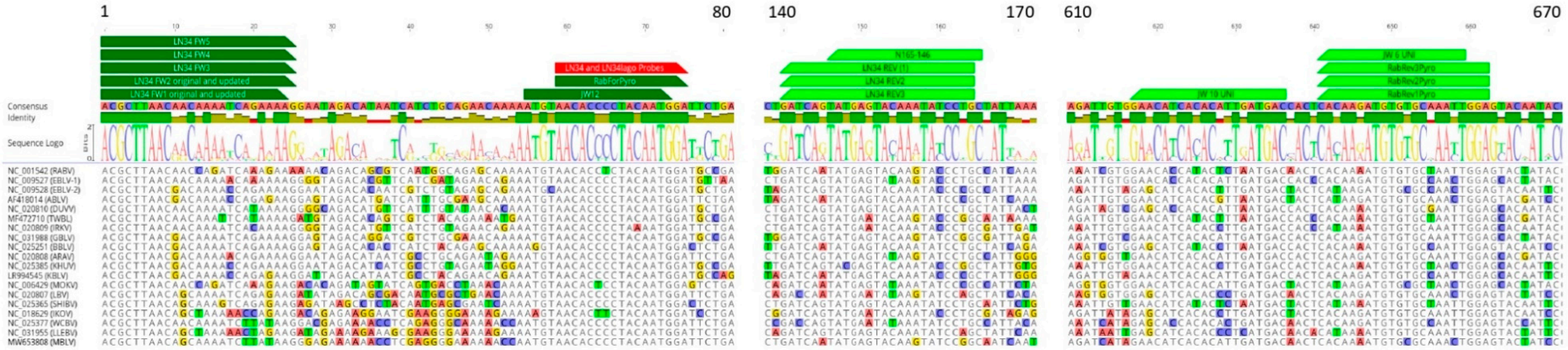

Figures were created using Geneious Prime® 2022.1.1 (Biomatters, Auckland, New Zealand). Dark green – forward primers, light green – reverse primers, red – probes.

Figure S1. The annealing regions of the oligonucleotide sets under evaluation demonstrated on representative sequences of all 18 known lyssavirus species

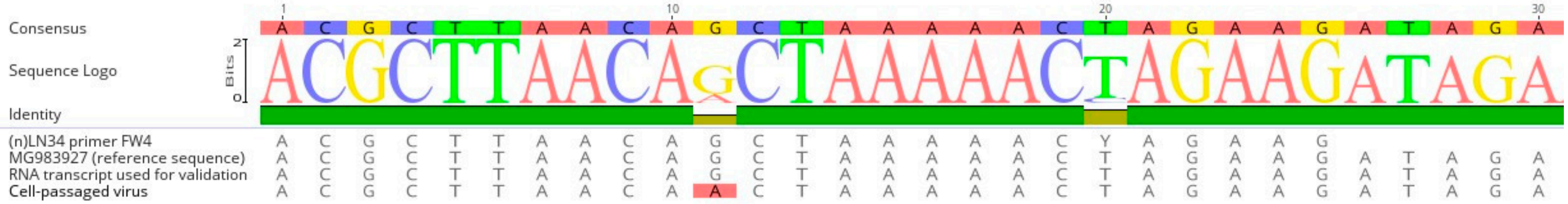

Figures were created using Geneious Prime® 2022.1.1 (Biomatters, Auckland, New Zealand). The virus sequences were obtained as published elsewhere [2] with minor modifications.

Figure S2. The nucleotide alignment of (n) LN34 FW4 primer, LLEBV reference sequence, LLEBV RNA transcript used for validation and cell-passaged LLEBV virus.
