# Supplementary material for: Comparison of Pan-Lyssavirus RT-PCRs and Development of an Improved Protocol for Surveillance of Non-RABV Lyssaviruses
Source: Viruses. 2023 Mar 4;15(3):680. doi: 10.3390/v15030680 (PMC10052027; doi:10.3390/v15030680)
Supplement: Supplementary file 1 [file viruses-15-00680-s001.zip › viruses-2175311-Supplementary Table S1.pdf]

Supplementary Table S1. The complete list of the sequences and the minimal number of mismatches observed between each primers/probes set and target sequence

Phylogroup	Species	Accession number	LN34			(n) LN34			JW12/N165-146		Hemi-nested RT-PCR				Convetional RT-PCR	
											1 round		2 round			
			FWs	Probes	REV	FWs	Probes	REVs	FW	REV	FW	REV	FW	REV	FW	REVs
I	ABLV	AF418014	2	0	2	0	0	1	0	2	0	1	0	1	0	1
I	ARAV	NC_020808	3	0	1	0	0	1	0	1	0	1	0	0	0	1
I	BBLV	LT839617	2	0	2	0	0	2	2	3	2	0	2	0	0	1
I	BBLV	LT839642	2	0	1	0	0	1	1	1	1	0	1	1	0	0
I	BBLV	LT839643	2	0	2	0	0	2	2	3	2	0	2	0	0	1
I	BBLV	LT839644	2	0	1	0	0	1	1	1	1	0	1	1	0	0
I	BBLV	MF043188	2	0	1	1	0	1	2	2	2	0	2	0	0	1
I	BBLV	NC_025251	2	0	1	0	0	1	1	1	1	0	1	1	0	0
I	DUVV	EU293120	1	0	1	0	0	1	0	1	0	0	0	0	0	0
I	DUVV	EU623444	1	0	1	0	0	1	0	1	0	0	0	0	0	1
I	DUVV	JN986749	0	0	1	0	0	1	0	1	0	0	0	1	0	0
I	DUVV	NC_020810	1	0	1	0	0	1	0	1	0	0	0	0	0	0
I	EBLV-1	EU293109	2	0	0	1	0	0	0	0	0	0	0	0	0	0
I	EBLV-1	EU293112	1	0	0	0	0	0	0	0	0	0	0	0	0	0
I	EBLV-1	EU626551	2	0	0	1	0	0	0	0	0	0	0	0	0	0
I	EBLV-1	EU626552	2	0	0	1	0	0	0	0	0	0	0	0	0	0
I	EBLV-1	KF155003	2	0	0	1	0	0	0	0	0	0	0	0	0	0
I	EBLV-1	KP241939	2	0	0	1	0	0	0	0	0	0	0	0	0	0
I	EBLV-1	LT839608	1	0	1	0	0	1	0	1	0	0	0	0	0	0
I	EBLV-1	LT839609	1	0	0	0	0	0	0	0	0	0	0	0	0	0
I	EBLV-1	LT839610	2	0	0	1	0	0	0	0	0	0	0	0	0	0
I	EBLV-1	LT839612	1	0	0	0	0	0	0	0	0	0	0	0	0	0
I	EBLV-1	LT839613	2	0	0	1	0	0	0	0	0	0	0	0	0	0
I	EBLV-1	LT839614	2	0	0	1	0	0	0	0	0	0	0	0	0	0
I	EBLV-1	MF187802	2	0	0	1	0	0	0	0	0	0	0	0	0	0
I	EBLV-1	MF187804	1	0	0	0	0	0	0	0	0	0	0	0	0	0
I	EBLV-1	MF187805	1	0	0	0	0	0	0	0	0	0	0	0	0	0
I	EBLV-1	MF187808	1	0	0	0	0	0	0	0	0	0	0	0	0	0
I	EBLV-1	MF187809	2	0	0	1	0	0	0	0	0	0	0	0	0	0
I	EBLV-1	MF187810	1	0	0	0	0	0	0	0	0	0	0	0	0	0
I	EBLV-1	MF187813	1	0	0	0	0	0	0	0	0	0	0	0	0	0
I	EBLV-1	MF187814	1	0	0	0	0	0	0	0	0	0	0	0	0	0
I	EBLV-1	MF187815	1	0	0	0	0	0	0	0	0	0	0	0	0	0
I	EBLV-1	MF187817	1	0	0	0	0	0	0	0	0	0	0	0	0	0
I	EBLV-1	MF187818	1	0	0	0	0	0	0	0	0	0	0	0	0	0
I	EBLV-1	MF187819	1	0	0	0	0	0	0	0	0	0	0	0	0	0
I	EBLV-1	MF187820	1	0	0	0	0	0	0	0	0	0	0	0	0	0
I	EBLV-1	MF187821	2	0	0	1	0	0	0	0	0	0	0	0	0	0

Phylogroup	Species	Accession number	LN34			(n) LN34			JW12/N165-146		Hemi-nested RT-PCR				Convetional RT-PCR	
											1 round		2 round			
			FWs	Probes	REV	FWs	Probes	REVs	FW	REV	FW	REV	FW	REV	FW	REVs
I	EBLV-1	MF187822	1	0	0	0	0	0	0	0	0	0	0	0	0	0
I	EBLV-1	MF187823	2	0	0	1	0	0	0	0	0	0	0	0	0	0
I	EBLV-1	MF187827	1	0	0	0	0	0	0	0	0	0	0	0	0	0
I	EBLV-1	MF187828	1	0	0	0	0	0	0	0	0	0	0	0	0	0
I	EBLV-1	MF187829	1	0	0	0	0	0	0	0	0	0	0	0	0	0
I	EBLV-1	MF187830	1	0	0	0	0	0	0	0	0	0	0	0	0	0
I	EBLV-1	MF187831	2	0	0	1	0	0	0	0	0	0	0	0	0	0
I	EBLV-1	MF187832	1	0	0	0	0	0	0	0	0	0	0	0	0	0
I	EBLV-1	MF187833	1	0	0	0	0	0	0	0	0	0	0	0	0	0
I	EBLV-1	MF187834	1	0	0	0	0	0	0	0	0	0	0	0	0	0
I	EBLV-1	MF187835	1	0	0	0	0	0	0	0	0	0	0	0	0	0
I	EBLV-1	MF187836	1	0	0	0	0	0	0	0	0	0	0	0	0	0
I	EBLV-1	MF187838	1	0	0	0	0	0	0	0	0	0	0	0	0	0
I	EBLV-1	MF187842	1	0	0	0	0	0	0	0	0	0	0	0	0	0
I	EBLV-1	MF187844	1	0	0	0	0	0	0	0	0	0	0	0	0	0
I	EBLV-1	MF187845	1	0	0	0	0	0	0	0	0	0	0	0	0	0
I	EBLV-1	MF187847	1	0	0	0	0	0	0	0	0	0	0	0	0	0
I	EBLV-1	MF187848	1	0	0	0	0	0	0	0	0	0	0	0	0	0
I	EBLV-1	MF187850	1	0	0	0	0	0	0	0	0	0	0	0	0	0
I	EBLV-1	MF187859	1	0	0	0	0	0	0	0	0	0	0	0	0	0
I	EBLV-1	MF187863	1	0	0	0	0	0	0	1	0	0	0	0	0	0
I	EBLV-1	MF187864	2	0	0	1	0	0	0	0	0	0	0	0	0	0
I	EBLV-1	MF187865	2	0	0	1	0	0	0	0	0	0	0	0	0	0
I	EBLV-1	MF187868	2	0	0	1	0	0	0	0	0	0	0	0	0	0
I	EBLV-1	MF187871	2	0	0	1	0	0	0	0	0	0	0	0	0	0
I	EBLV-1	MF187872	2	0	0	1	0	0	0	0	0	1	0	0	0	1
I	EBLV-1	MF187873	2	0	0	1	0	0	0	0	0	0	0	0	0	0
I	EBLV-1	MF187874	2	0	0	1	0	0	0	0	0	0	0	0	0	0
I	EBLV-1	MW551946	2	0	0	1	0	0	0	0	0	0	0	0	0	0
I	EBLV-1	NC_009527	2	0	0	1	0	0	0	0	0	0	0	0	0	0
I	EBLV-1	OU519345	2	0	0	1	0	0	0	0	0	0	0	0	0	0
I	EBLV-1	OU519351	2	0	0	1	0	0	0	0	0	0	0	0	0	0
I	EBLV-1	OU519352	2	0	0	1	0	0	0	0	0	0	0	0	0	0
I	EBLV-1	OU519358	2	0	0	1	0	0	0	0	0	0	0	0	0	0
I	EBLV-1	OU519369	2	0	0	1	0	0	0	0	0	0	0	0	0	0
I	EBLV-1	OU519370	2	0	0	1	0	0	0	0	0	0	0	0	0	0
I	EBLV-1	OU519371	2	0	0	1	0	0	0	0	0	0	0	0	0	0
I	EBLV-1	OU524412	2	0	0	1	0	0	0	0	0	0	0	0	0	0
I	EBLV-1	OU524414	2	0	0	1	0	0	0	0	0	0	0	0	0	0

Phylogroup	Species	Accession number	LN34			(n) LN34			JW12/N165-146		Hemi-nested RT-PCR				Convetional RT-PCR	
											1 round		2 round			
			FWs	Probes	REV	FWs	Probes	REVs	FW	REV	FW	REV	FW	REV	FW	REVs
I	EBLV-1	OU524415	2	0	0	1	0	0	0	0	0	0	0	0	0	0
I	EBLV-1	OU524419	2	0	0	1	0	0	0	0	0	0	0	0	0	0
I	EBLV-1	OU524421	2	0	0	1	0	0	0	0	0	0	0	0	0	0
I	EBLV-1	OU524423	2	0	0	1	0	0	0	0	0	0	0	0	0	0
I	EBLV-1	OU524424	2	0	0	1	0	0	0	0	0	0	0	0	0	0
I	EBLV-1	OU524427	1	0	0	0	0	0	0	0	0	0	0	0	0	1
I	EBLV-1	OU524428	2	0	0	1	0	0	0	0	0	0	0	0	0	0
I	EBLV-1	OU524429	2	0	0	1	0	0	0	0	0	0	0	0	0	0
I	EBLV-1	OU524431	2	0	0	1	0	0	0	0	0	0	0	0	0	0
I	EBLV-1	OU524432	2	0	0	1	0	0	0	0	0	0	0	0	0	0
I	EBLV-2	EU293114	3	0	0	0	0	0	1	0	1	0	1	0	0	0
I	EBLV-2	KF155004	3	0	0	0	0	0	1	0	1	0	1	0	0	0
I	EBLV-2	KY688132	3	0	0	0	0	0	1	0	1	0	1	0	0	0
I	EBLV-2	KY688133	3	0	0	0	0	0	1	0	1	0	1	0	0	0
I	EBLV-2	KY688134	3	0	0	0	0	0	1	0	1	0	1	0	0	0
I	EBLV-2	KY688135	3	0	0	0	0	0	1	0	1	0	1	0	0	0
I	EBLV-2	KY688136	3	0	0	0	0	0	1	0	1	0	1	0	0	0
I	EBLV-2	KY688137	3	0	0	0	0	0	1	0	1	0	1	0	0	0
I	EBLV-2	KY688138	3	0	0	0	0	0	1	0	1	0	1	0	0	0
I	EBLV-2	KY688139	3	0	0	0	0	0	1	0	1	0	1	0	0	0
I	EBLV-2	KY688140	3	0	0	0	0	0	1	0	1	0	1	0	0	0
I	EBLV-2	KY688141	3	0	0	0	0	0	1	0	1	0	1	0	0	0
I	EBLV-2	KY688142	3	0	0	0	0	0	1	0	1	0	1	0	0	0
I	EBLV-2	KY688143	3	0	0	0	0	0	1	0	1	0	1	0	0	0
I	EBLV-2	KY688144	3	0	0	0	0	0	1	0	1	0	1	0	0	0
I	EBLV-2	KY688145	3	0	0	0	0	0	1	0	1	0	1	0	0	0
I	EBLV-2	KY688146	3	0	0	0	0	0	1	0	1	0	1	0	0	1
I	EBLV-2	KY688147	3	0	0	0	0	0	1	0	1	0	1	0	0	0
I	EBLV-2	KY688148	3	0	0	0	0	0	1	0	1	0	1	0	0	0
I	EBLV-2	KY688149	3	0	0	0	0	0	1	0	1	0	1	0	0	0
I	EBLV-2	KY688150	3	0	0	0	0	0	1	1	1	0	1	0	0	0
I	EBLV-2	KY688151	2	0	0	1	0	0	1	0	1	0	1	0	0	0
I	EBLV-2	KY688152	3	0	0	0	0	0	1	0	1	0	1	0	0	0
I	EBLV-2	KY688153	3	0	0	0	0	0	1	0	1	0	1	0	0	0
I	EBLV-2	KY688154	3	0	0	0	0	0	1	0	1	0	1	0	0	0
I	EBLV-2	KY688155	3	0	0	0	0	0	1	0	1	0	1	0	0	0
I	EBLV-2	KY688156	3	0	0	0	0	0	1	1	1	0	1	0	0	0
I	EBLV-2	MG760848	3	0	0	0	0	0	1	0	1	0	1	0	0	0
I	EBLV-2	NC_009528	3	0	1	0	0	1	1	1	1	0	1	0	0	0

Phylogroup	Species	Accession number	LN34			(n) LN34			JW12/N165-146		Hemi-nested RT-PCR				Convetional RT-PCR	
											1 round		2 round			
			FWs	Probes	REV	FWs	Probes	REVs	FW	REV	FW	REV	FW	REV	FW	REVs
I	GBLV	KU244267	2	0	1	0	0	1	0	2	0	1	0	0	0	1
I	GBLV	KU244268	2	0	1	0	0	1	0	2	0	1	0	0	0	1
I	GBLV	KU244269	2	0	1	0	0	1	0	2	0	1	0	0	0	1
I	GBLV	NC_031988	2	0	1	0	0	1	0	2	0	1	0	0	0	1
I	IRKV	EF614260	2	1	0	1	1	0	1	1	1	1	1	1	1	1
I	IRKV	JX442979	3	1	0	2	1	0	1	1	1	1	1	1	1	0
I	IRKV	NC_020809	2	1	0	1	1	0	1	1	1	1	1	1	1	1
I	KBLV	LR994545	2	0	1	0	0	1	0	1	0	0	0	0	0	1
I	KHUV	NC_025385	3	0	1	0	0	1	0	2	0	0	0	1	0	1
I	RABV	NC_001542	0	0	0	0	0	0	0	0	0	0	0	0	0	0
I	RABV	OK317992	0	0	0	0	0	0	0	0	0	0	0	1	0	0
I	RABV	OK317995	0	0	0	0	0	0	0	0	0	0	0	1	0	0
I	RABV Africa	EU293113	0	0	0	0	0	0	0	1	0	0	0	0	0	1
I	RABV Africa	KC196743	0	0	0	0	0	0	0	0	0	0	0	1	0	0
I	RABV Africa	KF155001	0	0	0	0	0	0	0	1	0	0	0	0	0	0
I	RABV Africa	KF155002	0	0	0	0	0	0	0	0	0	0	0	1	0	1
I	RABV Africa	KF977826	0	0	1	0	0	1	0	1	0	0	0	0	0	0
I	RABV Africa	KP723638	0	0	1	0	0	1	0	2	0	0	0	0	0	1
I	RABV Africa	KR534220	0	0	0	0	0	0	0	0	0	0	0	1	0	1
I	RABV Africa	KR534232	0	0	0	0	0	0	0	0	0	0	0	1	0	1
I	RABV Africa	KR534234	0	0	0	0	0	0	0	0	0	0	0	1	0	0
I	RABV Africa	KR534246	0	0	0	0	0	0	0	0	0	0	0	1	0	1
I	RABV Africa	KR534254	0	0	0	0	0	0	0	0	0	0	0	1	0	1
I	RABV Africa	KR906743	0	0	0	0	0	0	0	0	0	0	0	1	0	0
I	RABV Africa	KR906745	0	0	0	0	0	0	0	0	0	0	0	1	0	1
I	RABV Africa	KR906747	0	0	0	0	0	0	0	0	0	0	0	1	0	0
I	RABV Africa	KR906748	0	0	0	0	0	0	0	0	0	0	0	0	0	0
I	RABV Africa	KR906751	0	0	0	0	0	0	0	0	0	0	0	1	0	0
I	RABV Africa	KR906752	0	0	0	0	0	0	0	0	0	0	0	0	0	0
I	RABV Africa	KR906756	0	0	0	0	0	0	0	0	0	0	0	1	0	1
I	RABV Africa	KR906765	0	0	0	0	0	0	0	0	0	0	0	1	0	1
I	RABV Africa	KR906779	0	0	0	0	0	0	0	0	0	0	0	1	0	0
I	RABV Africa	KT336433	0	0	0	0	0	0	0	0	0	0	0	1	0	0
I	RABV Africa	KT336435	0	0	0	0	0	0	0	0	0	0	0	1	0	0
I	RABV Africa	KT336437	0	0	0	0	0	0	0	0	0	0	0	1	0	0
I	RABV Africa	KU523255	0	0	0	0	0	0	0	1	0	0	0	0	0	1
I	RABV Africa	KX148100	0	0	0	0	0	0	0	1	0	0	0	0	0	1
I	RABV Africa	KX148101	0	0	0	0	0	0	0	0	0	0	0	0	0	0
I	RABV Africa	KX148107	0	0	0	0	0	0	0	0	0	0	0	0	0	0
I	RABV Africa	KY210261	0	0	0	0	0	0	0	0	0	0	0	1	0	0

Phylogroup	Species	Accession number	LN34			(n) LN34			JW12/N165-146		Hemi-nested RT-PCR				Convetional RT-PCR	
											1 round		2 round			
			FWs	Probes	REV	FWs	Probes	REVs	FW	REV	FW	REV	FW	REV	FW	REVs
I	RABV Africa	KY210265	0	0	0	0	0	0	0	0	0	0	0	1	0	1
I	RABV Africa	KY210268	0	0	0	0	0	0	0	0	0	0	0	1	0	1
I	RABV Africa	KY210284	0	0	0	0	0	0	0	0	0	0	0	1	0	0
I	RABV Africa	LC029889	0	0	0	0	0	0	0	0	0	0	0	1	0	1
I	RABV Africa	MG458307	1	0	2	0	0	2	0	2	0	0	0	1	0	1
I	RABV Africa	MG458318	0	0	0	0	0	0	0	0	0	0	0	1	0	0
I	RABV America North	AB645847	0	0	1	0	0	1	0	1	0	0	0	0	0	0
I	RABV America North	JQ685895	1	0	0	0	0	0	0	1	0	0	0	0	0	1
I	RABV America North	JQ685899	1	0	0	0	0	0	0	0	0	0	0	1	0	1
I	RABV America North	JQ685901	0	0	0	0	0	0	0	1	0	0	0	0	0	1
I	RABV America North	JQ685903	0	0	0	0	0	0	0	1	0	0	0	0	0	1
I	RABV America North	JQ685905	0	0	0	0	0	0	0	1	0	0	0	0	0	1
I	RABV America North	JQ685910	1	0	0	0	0	0	0	2	0	0	0	0	0	1
I	RABV America North	JQ685921	0	0	0	0	0	0	0	0	0	0	0	0	0	1
I	RABV America North	JQ685922	1	0	0	0	0	0	0	1	0	0	0	0	0	1
I	RABV America North	JQ685945	0	0	0	0	0	0	0	1	0	0	0	0	0	1
I	RABV America North	JQ685952	0	0	0	0	0	0	0	2	0	0	0	1	0	1
I	RABV America North	JQ685965	0	0	0	0	0	0	0	2	0	0	0	1	0	1
I	RABV America North	JQ685971	2	0	0	0	0	0	0	1	0	0	0	0	0	1
I	RABV America North	KU198460	0	0	0	0	0	0	0	0	0	0	0	0	0	0
I	RABV America North	KU198461	0	0	0	0	0	0	0	0	0	0	0	0	0	0
I	RABV America North	KU198472	0	0	0	0	0	0	0	0	0	0	0	0	0	0
I	RABV America North	KU198473	0	0	0	0	0	0	0	0	0	0	0	0	0	0
I	RABV America North	KU198479	0	0	0	0	0	0	0	0	0	0	0	0	0	0
I	RABV America North	KX036363	1	0	0	1	0	0	0	0	0	0	0	0	0	0
I	RABV America North	KX036366	1	0	0	1	0	0	0	0	0	0	0	0	0	0
I	RABV America North	KX148105	1	0	0	1	0	0	0	0	0	0	0	0	0	0
I	RABV America North	KY026417	0	0	0	0	0	0	0	1	0	0	0	0	0	1
I	RABV America North	KY026424	0	0	0	0	0	0	0	1	0	0	0	0	0	1
I	RABV America North	KY026427	0	0	0	0	0	0	0	1	0	0	0	0	0	1
I	RABV America North	MG458304	1	0	0	0	0	0	0	1	0	0	0	0	0	1
I	RABV America North	MK540739	0	0	0	0	0	0	0	1	0	0	0	0	0	1
I	RABV America North	MK540762	0	0	0	0	0	0	0	1	0	0	0	0	0	1
I	RABV America North	MK540821	0	0	0	0	0	0	0	1	0	0	0	1	0	1
I	RABV America South	AB362483	0	0	0	0	0	0	0	0	0	0	0	0	0	0
I	RABV America South	AB517659	0	0	0	0	0	0	0	0	0	0	0	0	0	0
I	RABV America South	AB519641	0	0	0	0	0	0	0	1	0	0	0	0	0	1
I	RABV America South	AB519642	1	0	0	1	0	0	0	1	0	0	0	0	0	1
I	RABV America South	EU293116	0	0	0	0	0	0	0	1	0	0	0	0	0	1
I	RABV America South	JQ685929	1	0	1	1	0	1	0	1	0	0	0	0	0	1

Phylogroup	Species	Accession number	LN34			(n) LN34			JW12/N165-146		Hemi-nested RT-PCR				Convetional RT-PCR	
											1 round		2 round			
			FWs	Probes	REV	FWs	Probes	REVs	FW	REV	FW	REV	FW	REV	FW	REVs
I	RABV America South	JQ685953	0	0	1	0	0	1	0	2	0	0	0	0	0	1
I	RABV America South	JQ685954	1	0	1	1	0	1	0	1	0	0	0	0	0	1
I	RABV America South	JQ685963	0	0	0	0	0	0	0	1	0	0	0	0	0	1
I	RABV America South	JQ685975	0	0	0	0	0	0	0	0	0	0	0	1	0	1
I	RABV America South	KX148109	0	0	1	0	0	1	0	2	0	0	0	0	0	1
I	RABV Asia	AB569299	0	0	0	0	0	0	0	1	0	0	0	0	0	1
I	RABV Asia	AB635373	0	0	0	0	0	0	0	1	0	0	0	0	0	1
I	RABV Asia	AB699220	0	0	0	0	0	0	0	0	0	0	0	0	0	0
I	RABV Asia	AB981663	0	0	1	0	0	1	0	1	0	0	0	0	0	1
I	RABV Asia	AB981664	0	0	1	0	0	1	0	1	0	0	0	0	0	1
I	RABV Asia	EF437215	1	0	0	1	0	0	0	0	0	0	0	0	0	0
I	RABV Asia	EU293111	0	0	1	0	0	1	0	1	0	0	0	0	0	1
I	RABV Asia	FJ712193	0	0	1	0	0	1	0	2	0	0	0	0	0	0
I	RABV Asia	GU345747	0	0	1	0	0	1	0	2	0	0	0	0	0	0
I	RABV Asia	GU345748	0	0	0	0	0	0	0	2	0	0	0	0	0	0
I	RABV Asia	HE802675	1	0	0	0	0	0	0	0	0	0	0	0	0	0
I	RABV Asia	HE802676	2	0	0	1	0	0	0	0	0	0	0	0	0	0
I	RABV Asia	JN609295	1	0	0	1	0	0	0	2	0	0	0	0	0	0
I	RABV Asia	JN786877	0	0	1	0	0	1	0	1	0	0	0	0	0	1
I	RABV Asia	JQ423952	1	0	1	1	0	1	0	2	0	0	0	0	0	0
I	RABV Asia	JQ647510	0	0	1	0	0	1	0	2	0	0	0	0	0	0
I	RABV Asia	JQ970481	0	0	1	0	0	1	0	2	0	0	0	0	0	0
I	RABV Asia	JX088694	0	0	2	0	0	0	0	2	0	0	0	0	0	0
I	RABV Asia	KC169986	0	0	1	0	0	1	0	2	0	0	0	0	0	1
I	RABV Asia	KC762941	1	0	1	1	0	1	0	2	0	0	0	0	0	1
I	RABV Asia	KF154998	0	0	0	0	0	0	0	0	0	0	0	0	0	0
I	RABV Asia	KF155000	1	0	1	0	0	1	0	1	0	0	0	0	0	0
I	RABV Asia	KF620487	0	0	1	0	0	1	0	3	0	0	0	0	0	1
I	RABV Asia	KF620488	0	0	1	0	0	1	0	3	0	0	0	0	0	1
I	RABV Asia	KF620489	0	0	1	0	0	1	0	3	0	0	0	0	0	1
I	RABV Asia	KF726852	1	0	1	1	0	1	0	3	0	0	0	0	0	1
I	RABV Asia	KF726853	1	0	1	1	0	1	0	2	0	0	0	0	0	0
I	RABV Asia	KJ004416	1	0	1	1	0	1	0	2	0	0	0	0	0	0
I	RABV Asia	KJ564280	0	0	1	0	0	1	0	2	0	0	0	0	0	0
I	RABV Asia	KM016899	0	0	0	0	0	0	0	0	0	0	0	0	0	0
I	RABV Asia	KY765901	0	0	0	0	0	0	0	0	0	0	0	0	0	0
I	RABV Europe	AY956319	1	0	0	1	0	0	0	0	0	0	0	0	0	0
I	RABV Europe	EU293115	0	0	0	0	0	0	0	0	0	0	0	0	0	0
I	RABV Europe	JQ944704	0	0	0	0	0	0	0	0	0	0	0	0	0	0
I	RABV Europe	JQ944705	0	0	0	0	0	0	0	0	0	0	0	0	0	0
I	RABV Europe	JQ944706	0	0	0	0	0	0	0	0	0	0	0	0	0	0

Phylogroup	Species	Accession number	LN34			(n) LN34			JW12/N165-146		Hemi-nested RT-PCR				Convetional RT-PCR	
											1 round		2 round			
			FWs	Probes	REV	FWs	Probes	REVs	FW	REV	FW	REV	FW	REV	FW	REVs
I	RABV Europe	JQ944707	1	0	0	1	0	0	1	0	1	0	1	0	0	0
I	RABV Europe	JQ944708	0	0	0	0	0	0	0	0	0	0	0	0	0	0
I	RABV Europe	KC595280	0	0	0	0	0	0	0	0	0	0	0	0	0	0
I	RABV Europe	KC595281	0	0	0	0	0	0	0	0	0	0	0	0	0	0
I	RABV Europe	KC595282	0	0	0	0	0	0	0	0	0	0	0	0	0	0
I	RABV Europe	KC595283	0	0	0	0	0	0	0	0	0	0	0	0	0	0
I	RABV Europe	KF154997	0	0	0	0	0	0	0	0	0	0	0	0	0	0
I	RABV Europe	KF154999	0	0	0	0	0	0	0	1	0	0	0	0	0	1
I	RABV Europe	KX148104	0	0	0	0	0	0	0	0	0	0	0	0	0	0
I	RABV Europe	LN879481	0	0	0	0	0	0	0	0	0	0	0	0	0	0
I	TBLV	MF472709	2	0	0	1	0	0	0	1	0	0	0	0	0	1
I	TBLV	MF472710	2	0	0	1	0	0	0	2	0	0	0	0	0	0
II	LBV	EU259198	1	0	2	1	0	2	0	3	0	1	0	1	0	1
II	LBV	EU293110	0	0	2	0	0	1	2	3	2	1	2	0	1	0
II	LBV	GU170202	1	0	1	1	0	1	0	1	0	1	0	0	0	3
II	LBV	JX901139	1	0	0	1	0	0	0	0	0	1	0	0	0	3
II	LBV	NC_020807	1	0	2	1	0	2	0	3	0	1	0	1	0	1
II	MOKV	EU293117	0	1	1	0	1	1	1	1	1	0	1	0	0	1
II	MOKV	EU293118	0	1	1	0	1	1	1	1	1	0	1	0	0	0
II	MOKV	KF155005	0	1	1	0	1	1	1	1	1	0	1	0	0	1
II	MOKV	KF155006	0	1	2	0	1	2	1	2	1	0	1	0	0	0
II	MOKV	KF155007	0	1	2	0	1	1	1	2	1	0	1	0	0	1
II	MOKV	NC_006429	0	1	2	0	1	2	1	2	1	0	1	0	0	0
II	SHIBV	NC_025365	2	0	1	2	0	1	0	1	0	0	0	0	0	1
III	IKOV	NC_018629	6	1	0	0	1	0	2	1	2	1	2	1	0	1
III	LLEBV	MG983927	5	0	2	0	0	0	0	2	0	1	0	2	0	1
III	LLEBV	NC_031955	5	0	2	0	0	0	0	2	0	1	0	2	0	1
III	MBLV	MW653808	3	0	1	1	0	1	0	2	0	1	0	0	0	2
III	WCBV	MZ501949	2	0	1	0	0	0	0	2	0	0	0	0	0	1
III	WCBV	NC_025377	2	0	1	0	0	0	0	2	0	0	0	0	0	1
