# Supplementary material for: Comparison of Pan-Lyssavirus RT-PCRs and Development of an Improved Protocol for Surveillance of Non-RABV Lyssaviruses
Source: Viruses. 2023 Mar 4;15(3):680. doi: 10.3390/v15030680 (PMC10052027; doi:10.3390/v15030680)
Supplement: Supplementary file 1 [file viruses-15-00680-s001.zip › viruses-2175311-Supplementary Table S2.pdf]

Supplementary Table S2. Analytical sensitivity (ASe) of LN34 rRT-PCR, the improved LN34 rRT-PCR, JW12/N165-146 rRT-PCR, hemi-nested RT-PCR, and one-step RT-PCR using RNA transcripts Limit of Detection (LoD; in bold) was determined as the higher dilution at which all tested replicates yielded positive results. For LN34 and (n) LN34, Ct values up to 35.9 were accepted. LoD determination for the JW12/N165-146 was based also on the presence of peaks with expected melting temperature.

RNA copies in µl	Phylogroup I																				Phylogroup II										Phylogroup III					
	RABV COSM		RABV AF2		EBLV-1b		EBLV-1a		EBLV-2		ABLV		ARAV		BBLV		KBLV		DUVV		LBV C		LBV A		MKV		SHIV		WCV		LLEBV		IKOV		MBLV	
	Mean	*SD*	*Mean*	*SD*	*Mean*	*SD*	*Mean*	*SD*	*Mean*	*SD*	*Mean*	*SD*	*Mean*	*SD*	*Mean*	*SD*	*Mean*	*SD*	*Mean*	*SD*	*Mean*	*SD*	*Mean*	*SD*	*Mean*	*SD*	*Mean*	*SD*	*Mean*	*SD*	*Mean*	*SD*	*Mean*	*SD*	*Mean*	*SD*
LN34																																				
**1E+08**																															31.6	0.2				
**1E+07**			14.2	0.93									24.8	0.44					17.4	0.28			15.3	0.10	16.4	0.2	17.5	0.2			**34.0**	**0.8**	27.5	0.2	27.5	0.3
**1E+06**	18.3	0.23	17.3	0.22	18.8	0.53							28.6	0.36					21.1	0.29	23.9	0.2	18.5	0.27	21.3	1.9	20.7	0.3	23.8	0.5	negative		30.4	0.5	30.5	0.4
**1E+05**	21.3	0.23	20.3	0.04	21.9	0.37			26.5	0.32	24.6	0.32	31.8	0.75			23.4	0.20	25.0	0.11	27.3	0.2	21.5	0.09	24.0	0.2	24.1	0.2	27.1	0.5	negative		**34.5**	**0.8**	**34.3**	**1.0**
**1E+04**	24.8	0.18	24.3	0.36	25.1	0.62			30.1	0.26	28.3	0.78	**34.7**	**0.68**	26.7	0.05	27.4	0.12	27.8	0.35	30.5	0.2	24.6	0.16	27.1	0.6	27.5	0.2	29.4	1.0	negative		negative		negative	
**1E+03**	28.0	0.39	27.3	0.41	28.1	0.25	29.8	0.41	**33.3**	**0.18**	31.0	0.79	38.9	1.38	30.7	0.44	30.8	0.88	31.1	0.30	**33.7**	**0.3**	28.3	0.13	30.4	0.5	31.4	0.9	**32.9**	**0.5**	negative		negative		negative	
**100**	31.4	0.35	30.5	0.55	31.7	0.46	**33.1**	**0.70**	negative		**34.6**	**0.65**	negative		**34.7**	**0.30**	**34.2**	**1.15**	**35.0**	**0.46**	37.3	0.8	31.9	0.32	**33.9**	**0.4**	**35.1**	**0.6**	36.2	0.6	negative					
**10**	**34.7**	**0.81**	**33.9**	**0.83**	**35.0**	**0.99**	36.5	0.80	negative		negative				negative		negative		negative		negative		**35.0**	**0.37**	negative		negative		negative		negative					
**1**	negative		negative		negative		negative		negative		negative				negative				negative		negative		negative		negative		negative		negative		negative					
(n) LN34																																				
**1E+08**																																				
**1E+07**																																				
**1E+06**																																				
**1E+05**													22.5	0.78			22.8	0.02																		
**1E+04**							24.8	0.26	24.5	0.11			25.4	0.19	25.4	0.05	26.7	0.14			29.3	0.04	26.1	1.42									24.8	0.51		
**1E+03**	28.6	0.25	26.4	0.09			28.5	0.13	28.0	0.16	29.3	0.71	28.7	0.08	29.7	0.19	29.9	0.92	27.7	0.18	**32.4**	**0.82**	29.0	0.74	28.3	0.21	**32.7**	**0.37**			29.1	0.3			30.7	0.75
**100**	30.7	0.46	29.6	0.09	31.2	0.63	**32.2**	**0.52**	31.4	0.40	**33.0**	**0.15**	**32.7**	**0.13**	**33.4**	**0.15**	**32.5**	**0.10**	31.4	0.23	negative		**32.5**	**0.86**	**32.2**	**0.71**	negative		**31.3**	**0.2**	**32.5**	**0.1**	**32.7**	**0.70**	**34.3**	**1.17**
**10**	**33.7**	**0.31**	**33.0**	**0.32**	**34.1**	**0.93**	negative		**35.0**	**0.29**	negative		negative		negative		negative		**34.2**	**0.33**	negative		negative		negative		negative		36.0	0.5	negative		negative		negative	
**1**	negative		negative		negative		negative		negative		negative		negative		negative		negative		negative		negative				negative		negative		negative		negative		negative		negative	
JW12/N165-146																																				
**1E+08**																																				
**1E+07**																					negative		28.3	0.44												
**1E+06**																					negative		33.3	1.22									30.1	0.29		
**1E+05**											**35.1**	**1.08**	24.2	0.74	21.2	0.11	23.7	0.17	30.6	0.40	negative		**36.9**	**0.65**					30.9	0.53			**34.4**	**0.50**	20.7	0.23
**1E+04**									29.9	0.38	negative		27.9	0.52	25.8	0.15	27.5	0.08	34.5	0.25	negative		negative		**37.3**	**0.90**			**37.1**	**1.74**	27.8	0.27	negative		25.1	0.47
**1E+03**	29.6	0.13	28.0	0.1	29.5	0.69	31.2	0.06	**33.3**	**0.43**	negative		32.8	1.08	29.6	0.43	31.5	0.40	**37.8**	**0.67**	negative		negative		negative		31.2	0.82	negative		31.7	0.41	negative		32.3	6.11
**100**	33.6	0.10	31.8	0.2	34.0	0.22	**35.4**	**0.33**	negative		negative		**35.6**	**0.48**	32.6	0.67	**34.1**	**0.60**	negative		negative		negative		negative		**34.2**	**0.61**	negative		**35.2**	**0.85**			33.0	0.22
**10**	**35.6**	**1.80**	**35.7**	**1.2**	**37.0**	**1.52**	negative		negative		negative		negative		**37.3**	**0.29**	negative		negative		negative		negative		negative		negative				negative				**36.0**	**0.31**
**1**	negative		negative		negative		negative		negative		negative		negative		negative		negative		negative				negative		negative		negative				negative				negative	
**Tm (°C)**	77.0		77.0		77.0		78.0		77.0		77.0		78.0		77.5		77.0		77.5				77.5		78.0		77.0		77.0		77.0		77.0		78.0	
hemi-nested RT-PCR: 1st round																																				
**1E+08**																																				
**1E+07**																					positive															
**1E+06**	positive														positive						**positive**										positive		positive			
**1E+05**	**positive**				positive								**positive**		**positive**		positive				negative						positive		positive		**positive**		**positive**			
**1E+04**	negative				positive								negative		negative		**positive**				negative						**positive**		**positive**		negative		negative			
**1E+03**	negative				**positive**		negative		negative		**positive**		negative		negative		negative		negative		negative		negative		negative		negative		negative		negative		negative		**positive**	
**100**	negative		negative		negative		negative		negative		negative		negative		negative		negative		negative		negative		negative		negative		negative		negative		negative		negative		negative	
**10**	negative		negative				negative		negative		negative		negative		negative		negative		negative		negative		negative		negative		negative		negative		negative		negative		negative	
**1**	negative		negative																										negative							
hemi-nested RT-PCR: 2nd round																																				
**1E+08**																																				
**1E+07**																					positive															
**1E+06**	positive														positive						**positive**										positive		positive			
**1E+05**	**positive**				positive								positive		**positive**		positive				negative						positive		positive		**positive**		**positive**			
**1E+04**	negative				positive								**positive**		negative		**positive**				negative						**positive**		positive		negative		negative			
**1E+03**	negative				**positive**		**positive**		**positive**		**positive**		negative		negative		negative		**positive**		negative		**positive**		**positive**		negative		**positive**		negative		negative		positive	
**100**	negative		**positive**		negative		negative		negative		negative		negative		negative		negative		negative		negative		negative		negative		negative		negative		negative		negative		**positive**	
**10**	negative		negative				negative		negative		negative		negative		negative		negative		negative		negative		negative		negative		negative		negative		negative		negative		negative	
**1**	negative		negative																										negative							
one-step RT-PCR																																				
**1E+08**																																				
**1E+07**																					**positive**										positive		positive			
**1E+06**													positive								negative								positive		positive		positive			
**1E+05**			positive		positive		positive		positive		positive		positive		positive		positive		positive		negative		positive		positive		positive		positive		positive		positive		positive	
**1E+04**			positive		positive		positive		positive		positive		positive		positive		positive		positive		negative		positive		**positive**		positive		positive		positive		positive		positive	
**1E+03**	positive		positive		**positive**		**positive**		positive		**positive**		positive		positive		**positive**		**positive**		negative		positive		negative		positive		positive		**positive**		**positive**		**positive**	
**100**	**positive**		**positive**		negative		negative		**positive**		negative		**positive**		**positive**		negative		negative		negative		**positive**		negative		positive		**positive**		negative		negative		negative	
**10**	negative		negative		negative		negative		negative		negative		negative		negative		negative		negative		negative		negative		negative		**positive**		negative		negative		negative		negative	
**1**	negative		negative		negative		negative		negative				negative		negative		negative		negative		negative		negative		negative		negative		negative		negative				negative	
