# Supplementary material for: Comparison of Pan-Lyssavirus RT-PCRs and Development of an Improved Protocol for Surveillance of Non-RABV Lyssaviruses
Source: Viruses. 2023 Mar 4;15(3):680. doi: 10.3390/v15030680 (PMC10052027; doi:10.3390/v15030680)
Supplement: Supplementary file 1 [file viruses-15-00680-s001.zip › viruses-2175311-Supplementary Table S3.pdf]

Table S3. Comparison of unique primer sequences created by combination of primers with low number of degenerated sites *Vs.* one primer including all degenerations

**Name**	**S/As**	**Sequence (5′ to 3′)**	**Length (nt)**	**Number of unique primer sequences**
FW1	S	ACGCTTAACAAC**M**A**R**ATCAAAGAA	24	4
FW2	S	ACGCTTAACAACAAAATCA**D**A**R**AAG	25	6
FW3	S	ACGCTTAACGACAAAA**H**CAGA**R**AAG	25	6
FW4	S	ACGCTTAACAGCTAAAAAC**Y**AGAAG	25	2
FW5	S	ACGCTTAACA**R**CAAAATCTTATAAG	25	2
Total number of unique primer sequences using 5 FW primers with low number of degenerations				**20**
Hypothetical FW primer using one highly degenerated primer	S	ACGCTTAAC**RR**C**H**A**R**A**HMHN**A**D**AA	24	**5184**
REV1*	As	C**M**GGGTA**Y**TTRTA**Y**TCATA**Y**TG**R**TC	25	64
REV2	As	CTGGATATTTGTA**Y**TCATA**Y**TGATC	25	4
REV3	As	CAGGATATTTATATTCATACTGGTC	25	1
Total number of unique primer sequences using 3 REV primers with low number of degenerations				**69**
Hypotetical REV primer using one highly degenerated primer	As	C**M**GG**R**TA**Y**TTRTA**Y**TCATA**Y**TG**R**TC	25	**128**
Probe1*	S	AACACC**Y**CTACAATGGA	17	2
Probe2*	S	AACACTACTACAATGGA	17	1
Total number of unique primer sequences using 2 probes with low number of degenerations				**3**
Hypothetical Probe using one highly degenerated probe	S	AACAC**YY**CTACAATGGA	17	**4**

Oligonucleotides with * are identical to original protocol. S: sense primers, As: antisense primers. Number of uniqu primer sequences generated by “primer3” in Geneious Prime® 2022.1.1 (Biomatters, Auckland, New Zealand. Degenerated sites are shown **in bold**.
